# Comparing laparoscopic cholecystectomy in patients with chronic obstructive pulmonary disease under spinal anesthesia and general anesthesia

**DOI:** 10.1186/s12893-018-0396-1

**Published:** 2018-08-20

**Authors:** Mehmet Bayrak, Yasemin Altıntas

**Affiliations:** 1 Ortadogu Hospital, 01360 Adana, Turkey; 2 Ortadogu Hospital, Ziyapasa mahallesi 67055 sokak no:1, Adana, Turkey

**Keywords:** COPD, Cholecystectomy, Laparoscopic, Regional anesthesia, Spinal anesthesia

## Abstract

**Background:**

Epidemiological data demonstrate that the worldwide prevalence of chronic obstructive pulmonary disease is increasing. These patients have an increased risk of mortality and morbidity and have constant limitations in airflow. Comparing laparoscopic cholecystectomy (LC) in patients with chronic obstructive pulmonary disease (COPD) under spinal anesthesia (SA) and general anesthesia (GA).

**Methods:**

We prospectively evaluated COPD patients who underwent laparoscopic cholecystectomy under general anesthesia (Group 1, *n* = 30) or spinal anesthesia (Group 2, n = 30) in our clinic between January 2016 and January 2018. Patients with COPD were further divided into groups according to their preoperative stages (Stage 1–4). Intraoperative vital findings, postoperative pain, complications, and length of hospitalization were compared between the general (GA) and spinal anesthesia (SA) groups.

**Results:**

The mean age of the patients in the GA group was 61.0 ± 6.7 years and was 61.0 ± 7.7 years in the SA group. In the GA and SA groups, the mean ASA score was 2.8 ± 0.6 and 2.9 ± 0.6, respectively, the mean operation duration was 31.7 ± 5.1 and 30.6 ± 5.1 min, respectively, and the length of hospitalization was 3.2 ± 1.7 and 1.5 ± 0.5 days, respectively. The partial carbon dioxide rates (PaCO2) at the postoperative 5th and 20th minutes were lower in the SA group than in the GA group. Further, the requirement for postoperative analgesia was lower in the SA group, and the length of hospitalization was significantly shorter in the SA group. There was no significant difference between the two groups in terms of operation duration.

**Conclusion:**

Laparoscopic cholecystectomy is a rather safe procedure for COPD patients under general and spinal anesthesia. However, spinal anesthesia is preferred over general anesthesia as it has better postoperative analgesia and causes no impairment of pulmonary functions.

**Electronic supplementary material:**

The online version of this article (10.1186/s12893-018-0396-1) contains supplementary material, which is available to authorized users.

## Background

Patients with chronic obstructive pulmonary disease (COPD) have an increased risk of mortality and morbidity and have continuous limitations in airflow. These limitations are diagnosed by taking the ratio of forced expiratory volume in 1 s (FEV1) to forced vital capacity (FVC). The severity of limitations in airflow is determined solely by a patient’s FEV1, which has been shown to predict COPD patient mortality [1].

Several meta-analyses and systematic reviews have reported an increasing worldwide prevalence of COPD that can be attributed to smoking, increased life expectancy, and less active lifestyles. For adults older than 40 years, COPD’s worldwide prevalence (as defined physiologically) is ~9–10% [2, 3]. This high prevalence is putting a burden on both surgeons and anesthesiologists, who are both seeing an increase in the volume of high-risk respiratory patients. These high-risk patients who are undergoing endoscopic procedures, especially those with COPD, have unique issues that require careful consideration by anesthesiologists. Currently, general anesthesia (GA) is used for the majority of laparoscopic procedures. However, more recent studies have indicated that regional anesthesia (RA) may be a better choice for certain patients [4].

Laparoscopic cholecystectomy (LC) was approved for use in 1988, and since then, it has been used as the gold standard treatment method for symptomatic cholelithiasis [5]. Patients undergoing LC are typically given GA and are intubated endotracheally in order to prevent respiratory embarrassment and aspiration due to pneumoperitoneum. However, recent studies have revealed that RA may be more beneficial than GA in these patients [4, 6–8].

During LC, insufflation with carbon dioxide (CO2) can cause pneumoperitoneum, in which the venous pressure is lower than the intra-abdominal pressure (IAP), preventing the resorption of CO2 and causing hypercapnia. Respiratory effects of pneumoperitoneum can be reduced lung volume, increased peak airflow pressure, and reduced pulmonary compliance [3, 9].

COPD severity should be assessed with a preoperative exam, including a comprehensive medical history, spirometry, and measurement of arterial blood gas. It is well known that advanced COPD patients have adverse outcomes when given GA with tracheal intubation and intermittent positive pressure ventilation (IPPV). These patients may have an increased rate of pulmonary complications following surgery, and are more susceptible to hypoxemia, laryngospasm, barotrauma, bronchospasm, and cardiovascular instability. Therefore, many believe that RA is a better choice than GA for these patients. Additionally, it should be noted that spinal and epidural anesthesia (when given at the lumbar level) do not affect respiratory function [7, 10–12].

In our clinic, laparoscopic cholecystectomy (LC) is generally performed under GA. However, we recently performed LC under SA for patients who could not tolerate GA, including COPD patients undergoing upper abdominal surgery. Therefore, in the current study, we analyzed the outcomes of COPD patients who underwent LC with GA and SA.

## Methods

Patients with COPD admitted for LC were given the choice of spinal anesthesia instead of general anesthesia. The choice of different anesthesia was part of our routine standard care. Spinal anesthesia is a commonly used method in both laparoscopic cholecystectomy and other upper abdominal operations in our clinic. All volunteers provided informed consent. Written approval was obtained from Cukurova University Faculty of Medicine Clinical Ethical Board. 30 COPD patients had LC under GA (Group 1) and another 30 COPD patients had LC under SA (Group 2). The age, gender, ASA scores, preoperative spirometer findings (FEV1, FEV1/FVC), concomitant systemic diseases (hypertension, diabetes mellitus, coronary artery disease) were evaluated. Exclusion criterias for spinal anesthesia was upper abdominal surgery and body mass index (BMI) over 55.

The spirometry test records from every patient were used to determine COPD staging [10] and ASA scores (American Society of Anesthesiology).

During surgery, every patient’s vitals were monitored and recorded, including invasive systemic blood pressure, peripheral oxygen saturation (SpO2) via pulse oximetry, and electrocardiography. Following Allen’s test, PaCO2 was measured by right or left radial artery cannulation.

Group 1 (the GA group) received 0.6 mg/kg rocuronium bromide, 2 mg/kg propofol, and 2 μg/kg fentanyl. After 90-120s 100% oxygenation, these patients underwent orotracheal intubation. Maintenance of anesthesia was performed using 1 – 3% sevoflurane in 50% nitrous oxide/50% oxygen.

Group 2 (the SA group) received their anesthesia while sitting. First, the patients were injected with 1% xylocaine, and then they underwent lumbar puncture with a 25 gauge needle in the L2-L3 intervertebral space. Then, they were given an intrathecal injection of 25 mg fentanyl and 3ml hyperbaric bupivacaine (0.5%), following which they were told to lay supine for 5 minutes. LC was performed when the patient achieved a sensory level of T4 dermatome.

Patients who did not immediately reach the T4 level were put in a Trendelenburg position, and they were monitored every 5 minutes until the T4 level was reached. The surgery commenced as soon as the patient reached the T4 dermatome level. Ephedrine 5mcg was given in incremental intermittent IV boluses in order to stop the mean arterial blood pressure (MAP) from dropping more than 20% under the pre-anesthetic level. A fentanyl bolus of 25 mcg was repeated at 5 minute intervals for shoulder pain (max 50 mcg). We recorded all incidents of intraoperative nausea/vomiting, hypotension, and right shoulder pain.

Each patient was put in the supine, reverse Trendelenburg position with a right lateral tilt up with his/her arms fully abducted. Patients were tilted the smallest amount possible in order to facilitate exposure of their gallbladders (i.e., we minimally used the reverse Trendelenburg position and right shoulder elevation). A pressure of 10-12 mmHg was used for pneumoperitoneum, and a flow rate of 2L/min (gradually increased to 5L/min) was used for the initial insufflation of CO2. LC was performed using a standard three-trocar technique with a zero-degree optical scope. Gallbladder dissection began at the triangle of Calot with the identification and clipping of both the artery and cystic duct, followed by mobilization from the liver bed. A subhepatic drain was inserted after the gallbladder was removed.

The preoperative values of SpO2, arterial oxygen saturation (SaO2), PaCO2, and arterial partial oxygen pressure (PaO2) were recorded for each patient. PaCO2 was recorded 5 min following anesthesia induction and at 5 and 20 min of insufflation. PaCO2 was also measured

Five min following extubation and 20 min postoperatively. The duration of operation was recorded for each patient.

### Statistical method

The SPSS 17.0 packet program was used to analyze the data. Categorical measurements were summarized as numbers and percentages, and continuous measurements were summarized as means and standard deviations (or as medians and minimums-maximums when applicable). Categorical variables were compared with the Chi square test or Fisher test. Distributions were controlled, and Student’s t test was used to compare continuous measurements between the groups. Repeated Measures Variance Analyses were used to determine changes in variables according to the groups over time. Values of *p* < 0.05 were considered significant.

## Results

Demographic characteristics of the patients are presented in Tables 1 and 2. Laparoscopic cholecystectomy was performed under spinal anesthesia (30 patients) or general anesthesia (30 patients). None of the patients in either group was transferred to open surgery.

**Table 1:** Distribution of demographic variables

	Anesthesia method				Total		
	General		Spinal				
	n	%	n	%	n	%	p
Gender							
M	20	66.7	22	73.3	42	70.0	0.779
F	10	33.3	8	26.7	18	30.0	
BMI	30	34.80 ± 5.41	30	33.47 ± 5.99	60	34.13 ± 5.70	0.369
Asa Score							
2	9	30.0	7	23.3	16	26.7	0.835
3	17	56.7	19	63.3	36	60.0	
4	4	13.3	4	13.3	8	13.3	
Non-COPD diseases							
None	5	16.7	7	23.3	12	20.0	0.214
DM	0	0.0	4	13.3	4	6.7	
HT	19	63.3	15	50.0	34	56.7	
HT- DM	5	16.7	2	6.7	7	11.7	
HT- DM CAD	1	3.3	1	3.3	2	3.3	
CAD- HT	0	0.0	1	3.3	1	1.7	
COPD stage							
1	12	40.0	13	43.3	25	41.7	0.962
2	14	46.7	13	43.3	27	45.0	
3	4	13.3	4	13.3	8	13.3

**Table 2:** Distribution of demographic variables

	Anesthesia method						
	General		Spinal		Total		
	Mean ± SD	Median (Min-Max)	Mean ± SD	Median (Min-Max)	Mean ± SD	Median (Min-Max)	p
Age	61.0 ± 6.7	61 (45–74)	61.6 ± 7.7	62 (44–78)	61.3 ± 7.1	62 (44–78)	0.734
Pre-op Fev1	71.3 ± 12.3	73 (45–85)	71.1 ± 13.5	74 (40–84)	71.2 ± 12.8	73 (40–85)	0.952

All of the LC patients well-tolerated the spinal anesthesia, and only 10 (33.3%) experienced postoperative shoulder pain. This pain was relieved by shoulder massage in 5 patients (16.7%). The other 5 patients (16.7%) required analgesia with 25-50 mcg fentanyl. Preoperative hypotension occurred in 2 patients (6.7%), and both returned to normal with ephedrine 5 mg. Bradycardia occurred in 1 patient (3.3%), and normocardia was maintained with 0.25 mg atropine. Urinary retention occurred in 2 patients (6.7%) in the postoperative period, and therefore, urinary catheter was inserted. One patient experienced postoperative headache, and none of the patients needed endotracheal entubation.

Of the patients who underwent general anesthesia (GA), 1 experienced postoperative hypotension (3.3%), which was recovered by 5 mg ephedrine. Tachycardia occurred in 2 patients, which was recovered by increasing the respiration rate in the anesthesia device. Four patients (13.3%) required postoperative mechanic ventilation due to hypercarbia and acidosis. Non-invasive continuous positive airway pressure (CPAP) was administered to 2 patients for 2 hours. Two patients with Stage 3 COPD required prolonged entubation; one of these patients was weaned after 8 hours, and the other after 12 hours.

**Fig. 1:**
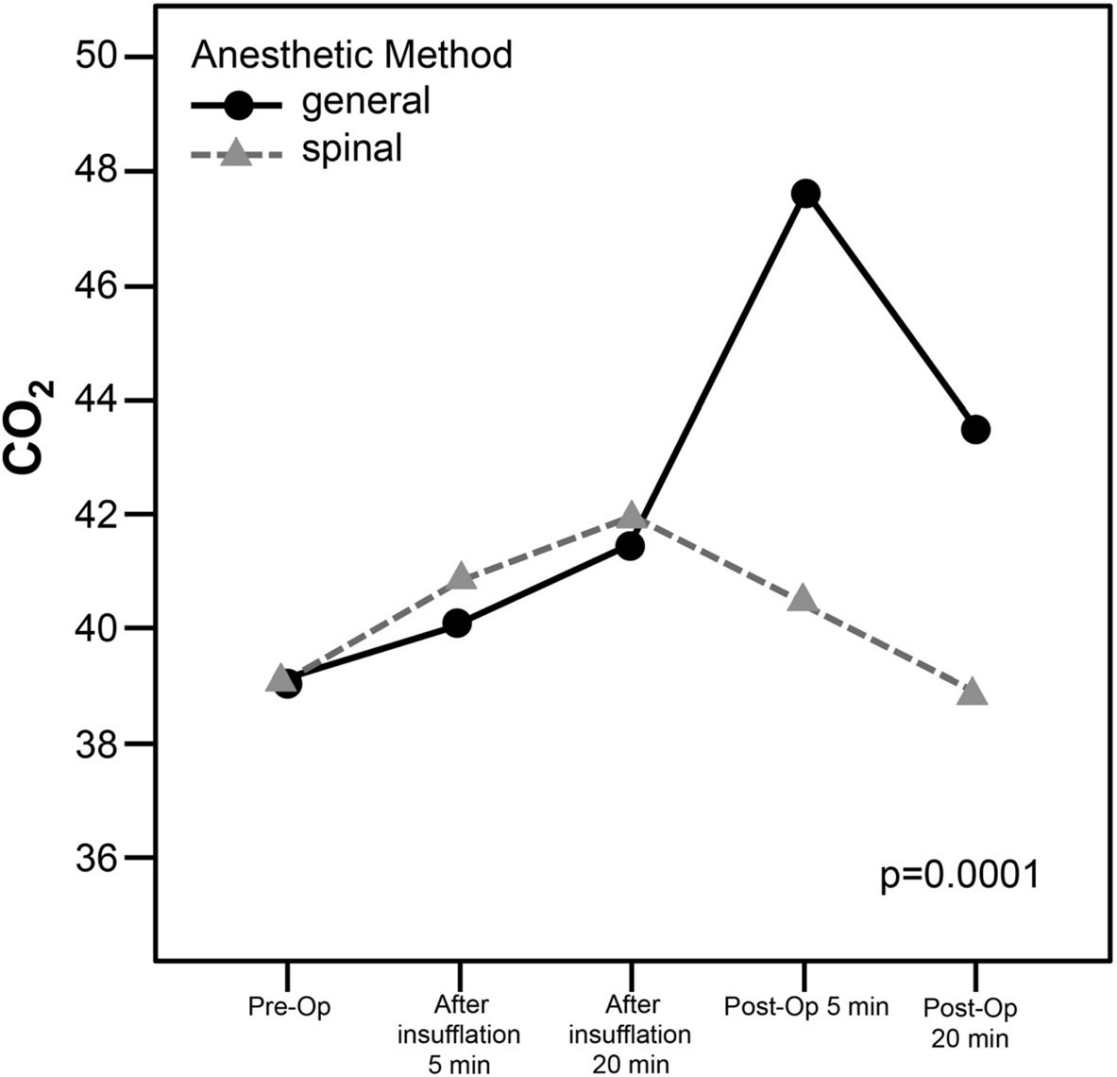
PaCO2 values for spinal and general anesthesia

PaCO2 values for both groups are shown in Fig. 1. No significant difference was determined in each group with regards to preoperative PaCO2 levels, or at the 5^th^ and 20^th^ minutes of insufflation. However, PaCO2 measurements were significantly higher in the general anesthesia group at the 5th and 20th postoperative minutes than in the SA group. In addition, the intergroup variation was significant with regards to the change in PaCO2 from the preoperative period to the 20th postoperative minutes (*p* = 0.0001).

The postoperative analgesia requirement is shown in Table 3. The postoperative analgesia requirement was significantly higher in the general anesthesia group.

**Table 3:** Postoperative analgesia

	Anesthesia method				Total		
	General		Spinal				
	n	%	n	%	n	%	p
Post-Op Analgesia, first 2 h							
Yes	30	100.0	0	0.0	30	50.0	0.0001
No	0	0.0	30	100.0	30	50.0	
Post-Op Analgesia between 2 and 8 h							
Yes	27	90.0	4	13.3	31	51.7	0.0001
No	3	10.0	26	86.7	29	48.3	

Postoperative morbidity is shown in Table 4. Although there was no significant difference between the GA and SA groups, postoperative pulmonary functions were impaired more frequently in the GA group, and 4 GA patients required mechanical ventilation.

**Table 4:** Postoperative morbidity

	Anesthesia method				Total		
	General		Spinal				
	n	%	n	%	n	%	p
Post-op Mechanical ventilation requirement							
Yes	4	13.3	0	0.0	4	6.7	0.122
No	26	86.7	30	100.0	56	93.3	
Post-op Shoulder pain							
Yes	4	13.3	10	33.3	14	23.3	0.489
No	26	86.7	20	67.7	46	76.7	
Post-Op Respiratory problem							
Yes	4	13.3	0	0.0	4	6.7	0.112
No	26	86.7	30	100.0	56	93.3	
Post-op Urine retention							
Yes	0	0	2	6.7	2	3.3	0.472
No	30	100.0	28	93.3	58	96.7	
Post-op Headache							
Yes	0	0	1	3.3	1	1.7	1.000
No	30	100.00	29	96.7	59	98.3	

There was no significant difference in operation duration between the SA and GA groups. However, the length of hospitalization was significantly lower in the SA group (Table 5).

**Table 5:** Comparison of groups related to operation duration and length of hospitalization

	Anesthesia method						
	General		Spinal		Total		
	Mean ± SD	Median (Min-Max)	Mean ± SD	Median (Min-Max)	Mean ± SD	Median (Min-Max)	p
Operation duration (min)	31.7 ± 5.1	33 (23–45)	30.6 ± 5.1	31 (22–41)	31.1 ± 5.1	32 (22–45)	0.318
Length of hospitalization (days)	3.2 ± 1.7	3 (2–10)	1.5 ± 0.5	2 (1–2)	2.4 ± 1.5	2 (1–10)	0.0001

## Discussion

Earlier studies have reported that following abdominal surgery, COPD patients have a significant risk of pulmonary complications [13–15]. The respiratory system is known to be affected by opiates, general anesthetic agents, mechanical ventilation, and myo- relaxants [16]. Anesthetics are known to cause changes in mucociliary transport; however, it is also known that abdominal surgery (in particular, upper abdominal surgery) can have unwanted negative effects on the respiratory system, including closing volume, vital capacity (VC), functional residual capacity (FRC), and tidal volume (TV) [17, 18]. Patients with COPD who are receiving anesthesia via inhalation or intravenously may have respiratory problems due to impaired mucociliary clearance. In addition, COPD patients may have respiratory problems due to CO2 pneumoperitoneum, which can cause diaphragm splinting systemic CO2 absorption. Therefore, the use of LC with newer anesthetic techniques (e.g., spinal, epidural blockage) may be a safer option for COPD patients [12]. Herein, we report the use general and spinal anesthesia for COPD patients undergoing laparoscopic cholecystectomy.

Several reports have shown that patients undergoing laparoscopy experience changes in their CO2 parameters. In a study including 30 patients who underwent LC under GA, Ozyuvacı E et al. found that these patients had a significantly higher postoperative PaCO2 when compared to preoperative levels [19]. Another study reported similar results [20]. Likewise, in our current study, the postoperative PaCO2 levels of the GA group were significantly higher than those in the SA group. In our current study, 4 patients in the GA group required mechanical ventilation due to postoperative hypercarbia and acidosis. On the other hand, patients in the SA group well-tolerated the procedure, and had no impairment in respiratory functions. The study by Gramatica et al. further supports the use of regional anesthesia (RA) for LC in advanced COPD cases, as they reported that all of the patients were stable throughout the surgery [11]. Likewise, in 1998, Pursnani et al reported that LC with RA could be safely used in severe COPD patients [12].

An important predictor of postoperative pulmonary function is effective analgesia. Since opioids can cause excessive sedation, spinal analgesia is a better choice, as it does not have this side effect and therefore lowers the risk of respiratory failure. Several studies have shown that RA can be useful for alleviating the postoperative pain of COPD patients [1, 11, 12, 19, 21, 22]. In our current study, results indicate that patients who underwent LC with SA had significantly less early analgesic requirement and postoperative pain compared to patients who underwent LC with GA; the SA patients had a reduction in pulmonary complications.

However, right shoulder pain is a complication of LC with SA. This pain is most likely due to the CO2 pneumoperitoneum irritating the diaphragm. Previous studies have shown that the incidence for intraoperative right shoulder pain (requiring iv fentanyl) was between10 and 55.2% [7, 8, 11, 23, 24]. In our current study, 10 (33.3%) SA patients reported shoulder pain; 5 (16.7%) of these required fentanyl, while 5 (16.7%) were satisfied with shoulder massage.

Results of our current study revealed no significant difference in the average operation time between the GA and SA groups. This is most likely because we utilized an experienced surgical team who kept the operative times very short.

Other studies reported mean operation duration times as 47.4 min (Kalaivani V. et al.) and as 40 min (Pursnani et al.) [12, 25].

In our current study, 2 patients (6.7%) in the SA group suffered from urinary retention; however, this complication did not increase their hospitalization times.

The mean length of the hospitalization was 1.5 ± 0.5 days in the SA group, which was significantly lower than that of the GA group (3.2 ± 1.7 days). It was determined that the SA patients had a faster recovery. A previous study by Mean Chi Hsun Hsieh et al. reported the mean length of the hospitalization to be 3.3 ± 1.6 dats in 20 patients with COPD who underwent LC with GA [21].

## Conclusion

We conclude that laparoscopic cholecystectomy can be performed safely under GA and SA in patients with COPD**.** However, we recommend that SA should be used in COPD patients, as it reduces the risk of extubation, bronchoconstriction and respiratory depression, and decreases the requirement for postoperative mechanical ventilation, leading to a faster postoperative recovery. We believe that this study can be supported by larger randomized trials.

## Additional file

Additional file 1: Regional and general anesthesia COPD patients. (XLSX 14 kb)

## Abbreviations

ASA: American society of anesthesiologists
ASA: American society of anesthesiologists
BMI: Body mass index
COPD: Chronic obstuctive pulmonary disease
FEV: Forced expitaroy volume
FVC: Forced vital capacity
GA: General anesthesia
IAP: Intra – abdominal pressure
IPPV: Intermittent positive pressure ventilation
L: Lumbar
L: Lumbar
LC: Laparoscopic cholecystectomy
MAP: Mean arterial blood pressure
NG: Nasogastric tube
Pa CO2: Partial carbon dioxide rates
RA: Regional anesthesia
SA: Spinal anesthesia
SaO2: Arterial oxygen saturation
SPO2: Blood oxygen saturation
T: Thoracal
T: Thoracic
VAS: Visual analogue scale
VC: Vital capacity
